# General Pathology

**Published:** 1895-12-21

**Authors:** 


### GENERAL PATHOLOGY.

(*Continued from page* 183.)

**Small-pox.**—The search for the infective organism to which the disease is due has produced papers by Jackson Clarke and S. Monckton Copeman. The former[1] describes the amœboid bodies which he has found after vaccination on the cornea of rabbits, as dense, highly refractile particles, which show a central spot, undergo segmentation, and break up into spores. These bodies are produced after vaccination, and not by the application of mechanical irritants. He finds them also in the skin and other parts of the cadaver of persons who have died of variola, and he regards them as akin to the bodies he has described in cancer. Copeman remarks that they have not been shown to have any causal relation to the disease; and in speaking of the bacilli which he and Klein found in the early stages of variola vesicles, announces[2] that he has been able to cultivate them in hen's eggs. These were inoculated under aseptic precautions, and kept at a temperature of 37 deg. In a month's time the bacilli had multiplied greatly, and from them calves were inoculated. Again, from the vesicular points which arose other calves were vaccinated, and finally Dr. Copeman himself and a number of children with complete success. These experiments are being repeated, that any accidental contamination by the ordinary vaccine (which was being used in the same place) may be eliminated; for it has been urged that the many cases of vaccination of cattle by variolous matter, and their subsequent yield of vaccinal matter are due to accidental contamination of the instruments with true vaccine. Copeman is therefore compelled to disprove this theory in regard to vaccination from his egg cultures, as he also claims to have done in some direct vaccinations of calves from variolous patients. Hlava and Houl[3] have tried to obtain a serum which may replace vaccine. The serum was obtained from calves which had been vaccinated once or several times. This serum injected into other calves and children prevented the development of vaccine vesicles if inoculation was performed some days later. With regard to so-called "well-vaccinated localities," W. S. Tebb maintained that the epidemics in several of them were greater than in Leicester and Keighley, but the *British Medical Journal* points out[4] that in Leicester probably 98 per cent. of the population were vaccinated, and the mortality among these cases was only 5 per cent. While in vaccinated children under 10 the risk of death from small-pox is very small indeed, the protection without re-vaccination undoubtedly diminishes with age. Moreover, in every community there is an unvaccinated or badly vaccinated section, among whom a severe attack of small-pox may cause great destruction. In the Bristol epidemic[5] the deaths among the unvaccinated cases were 26·8 per cent., and those among the vaccinated 6·6, only one being among the re-vaccinated, and none in protected children under 10. In Brighouse[6] the figures were 30·6 and 4·85, while there were no deaths among the re-vaccinated, and in four cases the fact of vaccination was uncertain. In Manchester, Niven remarks on the difficulty ordinary practitioners have of diagnosing small-pox. The mortality among his 996 cases was for unvaccinated 26·47 per cent., for the vaccinated 4·04, and among the doubtful 13·2; and no death in a vaccinated person under 15 occurred. The percentage of unvaccinated children in the country generally is increasing to an alarming extent owing to the delayed report of the Royal Commission. The Local Government Board find that no less than 13·4

per cent. are unaccounted for in 1891, the latest year reported.

**Malaria.**—In reply to Dr. Daniels, the editor of the *British Medical Journal*[7] acknowledges that the parasite is not always to be found at all times in every cycle of a malarial fever, and occasionally not at all during the first twenty-four hours, at least, in the peripheral circulation. But in the cases met with in this country which have remained latent for a long while since their removal from the tropics, Daniels[8] himself states that the amœba can be invariably detected.

**Cholera.**—Haffkine has now returned to England for his health, and we have at the same time reports from various quarters of his labours. The Medical Officer of Health for Calcutta[9] announces that 4,397 persons have been inoculated there, and that in 36 houses where inoculations took place, cholera made its appearance. Out of 521 persons in them the deaths of the uninoculated were 11·64 per cent., and only 2·2 per cent. of the inoculated. None of the latter, moreover, were completely inoculated by receiving the second injection five days after the other. In the Assam tea-gardens Haffkine carried out an enormous work, and even with a single inoculation there were only five cases and three deaths, while among the uninoculated there were 38 cases and 19 deaths. In consequence of these important results the Medical Officer asked the Corporation of Calcutta for 10,000 rupees for carrying on the the work. The Government[10] are passing a Bill for the better regulations of the Mecca pilgrim traffic, but have met with opposition in the Mussulman community on account of the stringent character of the regulations imposed. An even greater difficulty will be to ensure due precautions being taken in Turkish territory, especially during the present political difficulties. Cholera has, however, broken out at Damietta,[11] on one of the mouths of the Nile, but a good water supply has been made available. It was announced on November 4th that 266 cases and 148 deaths had occurred in Damietta, Mansoura, and Menzala.[12] However, the disease had not spread beyond its original seat, and energetic measures to combat it were being carried out. In Japan a frightful epidemic broke out on the return of the troops from the war, and in Seoul, the capital of Korea, some 6,000 persons died. T. H. Wells[13] reported valuable results there from the use of salol, with entero- and hypodermic injections of saline fluid. Two thousand persons have been attacked in Russia,[14] but at home we have fortunately escaped without any certain instance, for the proofs were wanting, that the cases at Grimsby and on the Humber were more than choleraic diarrhœa. Accounts of epidemics in places so far apart as Algiers[15] and Honolulu show, however, that the wave of infection is not exhausted.

[1] Lancet, Oct. 20. [2] Lancet, Aug. 10. [3] B. M. J., Oct. 26. [4] B. M. J., Nov. 9. [5] B. M. J., Sept. 28. [6] B. M. J., Sept. 14. [7] B. M J., Sept. 21. [8] B. M. J., Oct. 26. [9] B. M. J., Sept. 21. [10] B. M. J., Oct. 5. [11] Lancet, Oct. 26. [12] Lancet, Nov. 9. [13] Med. Rec., Oct. 5. [14] Lancet, Sept. 14. [15] Lancet, Sept. 21.

